# Commentary: The mental representation of integers: An abstract-to-concrete shift in the understanding of mathematical concepts

**DOI:** 10.3389/fpsyg.2018.00209

**Published:** 2018-02-27

**Authors:** Melinda A. Mende, Samuel Shaki, Martin H. Fischer

**Affiliations:** ^1^Division of Cognitive Sciences, Department of Psychology, University of Potsdam, Potsdam, Germany; ^2^Department of Behavioral Sciences, Ariel University, Ariel, Israel

**Keywords:** cognitive development, mental number line, negative numbers, embodied cognition, abstract concepts

Decision times during processing of positive number symbols (1, 2, 3 etc.) inform our understanding of mental representations of integers (Holyoak, 1978; Dehaene et al., 1993; Fischer and Shaki, 2014). Effects of number magnitude on cognition include *distance effects* (faster discrimination for larger numerical differences in a number pair), *size effects (faster processing of smaller numbers), Spatial-Numerical Association of Response Codes (SNARC;* faster left/right responses to small/large numbers), linguistic markedness (*MARC;* faster left/right responses to odd/even numbers) and *semantic congruity effects* (faster smaller/larger decisions over smaller/larger number pairs). Results converge on the notion of a spatially oriented mental number line (MNL) where numerically smaller number concepts exist to the left of larger number concepts. How do these performance signatures help us to understand the cognitive representation of negative number symbols (−1, −2, −3 etc.)? Unlike natural number symbols, negative number symbols lack corresponding real entities that support sensory-motor learning. We discuss a recent proposal by Varma and Schwartz (2011) with implications for developmental research.

## Terminological clarification

Different terms distinguish two fundamentally different views regarding the cognitive representation of negative numbers: The first view states that negative numbers are cognitively represented to the left of positive numbers, thereby extending the MNL infinitely leftward (henceforth called “extended MNL account”). The second view states that negative numbers have no cognitive representations but are understood through augmenting positive entries of the MNL (henceforth called “rule-based MNL account”). This dichotomy reflects identical distinctions made by Fischer (2003: ontogenetic vs. phylogenetic), Shaki and Petrusic (2005: extended number line vs. magnitude polarity), Ganor-Stern and Tzelgov (2008: holistic vs. components) and Varma and Schwartz (2011: analog+ vs. symbol+). Evidence from magnitude comparisons was used to support either account (see Table 1 for more studies) so we review it before recommending methodological improvements.

**Table 1:** Summary of previous empirical work on the cognitive representation of negative numbers.

**Task**	**Stimuli**	**Responses**	**Measures**	**References**
Magnitude comparison	Spatial	Spatial (discrete)	SNARC effect	Fischer, 2003
Magnitude comparison	Spatial	Spatial (discrete)	SNARC + Semantic congruity effect	Shaki and Petrusic, 2005
Magnitude comparison	Spatial	Spatial (discrete)	Distance effect + Semantic congruity effect	Ganor-Stern, 2012
Magnitude comparison	Spatial	Spatial (discrete)	Distance effect + Semantic congruity effect	Ganor-Stern et al., 2010
Magnitude comparison	Spatial	Spatial (discrete)	Semantic congruity effect + Size effect	Ganor-Stern and Tzelgov, 2008
Magnitude comparison	Spatial	Spatial (discrete)	Distance effect + Semantic congruity effect	Tzelgov et al., 2009
Magnitude comparison	Spatial	Spatial (discrete)	Sign-decade compatibility effect	Huber et al., 2015
Magnitude comparison	Spatial	Spatial (discrete)	Distance effect + Semantic congruity effect + Size effect	Varma and Schwartz, 2011
Magnitude comparison	Spatial	Spatial (discrete)	(Neural) Distance effect (fMRI)	Blair et al., 2012
Magnitude comparison	Spatial	Spatial (discrete)	(Neural) Distance effect (fMRI)	Gullick et al., 2012; Gullick and Wolford, 2013
Physical comparison	Spatial	Spatial (discrete)	Distance effect + Size Congruity Effect	Tzelgov et al., 2009
Physical comparison	Spatial	Spatial (discrete)	Congruity Effect (ERP)	Parnes et al., 2012
Magnitude comparison	Spatial	Spatial (discrete)	Number mining (fMRI)	Chassy and Grodd, 2012
Magnitude classification	Centered	Spatial (discrete)	Distance effect + SNARC	Krajcsi and Igács, 2010
Magnitude classification	Centered	Spatial (discrete)	SNARC effect	Fischer and Rottmann, (2005, Experiment 2)
Parity classification + Priming	Centered	Spatial (discrete)	SNARC effect	Tse and Altarriba, 2010
Parity classification	Centered	Spatial (discrete)	SNARC effect	Fischer and Rottmann (2005, Experiment 1)
Parity classification	Centered	Spatial (discrete)	SNARC effect	Nuerk et al., 2004
Parity classification	Centered	Spatial (discrete)	SNARC effect	Prather and Boroditsky, 2003
Pointing (Number line)	Centered	Spatial (continuous)	Scalar variability model	Ganor-Stern and Tzelgov, 2008
Pointing (Number line)	Centered	Spatial (continuous)	Linear or logarithmic	Young and Booth, 2015
Center Classification (Number line)	Spatial	Verbal	Leftward bias, SNARC effect	Loftus et al. (2009, Experiment 2)
Detection (visual)	Centered digit + Spatial target	Centered	Spatial shift of attention	Dodd, 2011
Detection (visual)	Centered digit + Spatial target	Centered	Spatial shift of attention	Zhang and You, 2012
Detection (auditory)	Centered digit + Spatial target	Centered	Spatial shift of attention	Kong et al., 2012

## Evidence from magnitude comparison

Magnitude comparison was first used by Fischer (2003) to report a cognitive processing signature for negative numbers: Adults identified the larger of two digits ranging from −9 to +9 and shown in pairs with constant numerical distance (to control both distance and MARC effects). Faster decisions obtained when the spatial arrangement of digits on screen matched a leftward-extended mental number line, thus supporting the extended MNL account. However, Shaki and Petrusic (2005) identified a confound with semantic congruity and showed that results depend on whether positive and negative numbers are blocked or mixed.

Ganor-Stern and Tzelgov (2008) found similar size effects for positive and negative numbers in the comparison task and a systematic decrease of localization variability with increasing number magnitude in a number-to-position task (where adults localized the position of numbers with a mouse cursor on a horizontal line). They inferred a rule-based MNL account.

Varma and Schwartz (2011) found an inverse distance effect in magnitude comparison with adults, inconsistent with a rule-based MNL which predicted no distance effect at all in mixed comparisons (with one positive and one negative integer), due to superficial sign comparisons. The authors augmented the extended MNL account by postulating additional knowledge about the relationship between positive and negative number concepts which is not available yet to 6th graders because they showed no inverse distance effect and thus used a rule-based MNL.

## Evidence from other methods

This conclusion is surprising, given the wide consensus for a concrete-to-abstract shift in knowledge development. Why are conclusions so heterogeneous, even when using a single task? Other methods assessed negative number representation, including pointing, parity judgments, brain activation, eye movement recording and computer simulation (see Table 1 for details). For example, Gullick and Wolford (2013) investigated neural distance effects in children. They found that IPS activity increased with age while parietal, frontal and precentral activity decreased, consistent with an anterior-posterior shift during maturation (Rivera et al., 2005). They concluded that practice and experience help to integrate negative numbers into an extended mental number line. In addition, Young and Booth (2015) found results both in line with an extended MNL and in line with a rule-based MNL account in two pointing experiments with middle school students. The authors concluded that this conflicting pattern could reflect under-developed number knowledge and differences in previous number exposure. In summary, previous findings in adult and children studies are highly controversial. The lack of consistent effects in adults does not provide a sufficient basis for firm developmental interpretations, thus distorting current conclusions about the development of negative number processing.

## Methodological comment

We believe that this ongoing debate benefits from a methodological comment. Specifically, we note that all published studies on negative number processing either presented spatially distributed stimuli or recorded response speed with lateralized keys (see Table 1). This use of spatially distributed stimuli or responses permits participants different strategies (e.g., selective attending to the sign or “mirroring” cf. Varma and Schwartz, 2011) and induces extraneous biases (e.g., the semantic congruity effect), all of which contaminates number processing (Fischer and Rottmann, 2005; Shaki and Petrusic, 2005; Gevers et al., 2010; Fischer and Shaki, 2016).

To address this concern, we recently developed a method where positive and negative numbers are interleaved with spatially oriented objects. Participants only ever see a single stimulus (number or object) and respond with a single button only if the relevant part of a conjunction rule was fulfilled (Fischer and Shaki, 2017). Examples are “respond only if the number is larger than −5 or the car is facing left” (incongruent rule) or “respond only if the number is smaller than−5 or the car is facing left” (congruent rule). We found that negative numbers are associated with space according to their signed magnitude, thus resolving the long-standing debate about the cognitive representation of negative numbers (Fischer, 2003; Shaki and Petrusic, 2005): Once the task prevents strategies, an extended mental number line prevails. This conclusion is based on results from a paradigm free of spatial or reporting biases. It can, in turn, inform our studies of the development of negative number concepts (Shaki and Fischer, 2018).

## Author contributions

All authors listed have made a substantial, direct and intellectual contribution to the work, and approved it for publication.

### Conflict of interest statement

The authors declare that the research was conducted in the absence of any commercial or financial relationships that could be construed as a potential conflict of interest.
